# Effects of Communicating Genetic Risk of Type 2 Diabetes and Wearable Technologies on Behavioral Outcomes in East Asians: Statistical Analysis Protocol for a Randomized Controlled Trial

**DOI:** 10.2196/65012

**Published:** 2025-11-05

**Authors:** Harrison Hin Sheung Ho, Ziyuan Chen, Job Godino, Michael Multhaup, Derwin King Chung Chan, Shiu Lun Au Yeung, Shan Luo, Brian Hon Yin Chung, Simon Griffin, Youngwon Kim

**Affiliations:** 1 School of Public Health, Li Ka Shing Faculty of Medicine The University of Hong Kong Hong Kong China (Hong Kong); 2 Herbert Wertheim School of Public Health & Human Longevity Science University of California San Diego La Jolla, CA United States; 3 Exai Bio Palo Alto, CA United States; 4 School of Arts and Social Sciences Hong Kong Metropolitan Universit Hong Kong China (Hong Kong); 5 Department of Paediatrics and Adolescent Medicine, Li Ka Shing Faculty of Medicine The University of Hong Kong Hong Kong China (Hong Kong); 6 Medical Research Council Epidemiology Unit, School of Clinical Medicine University of Cambridge Cambridge United Kingdom

**Keywords:** type 2 diabetes, physical activity, genetic risk, wearable technology, randomized controlled trial, movement behavior, statistical analysis plan

## Abstract

**Background:**

Evidence suggests that the communication of type 2 diabetes (T2D) genetic risk alone has limited effectiveness on facilitating behavioral changes among individuals of European descent. Although the use of wearable devices has been associated with changes in behavior, the effects of combining personalized precision medicine with wearable devices on behaviors related to T2D prevention remain unclear. This study aims to assess the novel effects of T2D genetic risk communication and wearable device functions on objectively measured moderate to vigorous physical activity (MVPA) time among East Asian individuals with overweight or obesity.

**Objective:**

The objectives of this study are to (1) investigate the effects of communicating T2D genetic risk and (2) examine the effects of combining T2D genetic risk communication with wearable device functions such as step goal setting and activity prompts on objectively measured MVPA time among East Asians with overweight or obesity.

**Methods:**

In this parallel-group randomized controlled trial, 355 East Asians with overweight or obesity aged between 40 and 60 years are allocated to 1 of 3 groups: 1 control and 2 intervention groups. Blood samples are used for estimation of T2D genetic risk and tested for metabolic risk markers. T2D genetic risk is estimated based on 113 single nucleotide polymorphisms associated with T2D among East Asians using an established method. All 3 groups receive a Fitbit device. Both intervention groups will receive T2D genetic risk estimates along with lifestyle advice, but one of the intervention groups will receive additional Fitbit functions: step goal setting and prompt functions. The intervention materials are delivered weekly using WhatsApp and monthly via email. The primary outcome is MVPA time, which is objectively measured with the built-in accelerometer of the Fitbit Inspire 3 and will be assessed at baseline, immediately after the intervention, 12 months after the intervention, and at 6-month follow-up. Secondary outcomes include other parameters, such as sedentary time, BMI, systolic and diastolic blood pressure, 5 metabolic risk markers, handgrip strength, sleep, activity calories, self-reported physical activity, self-reported fruit and vegetable consumption, smoking status, and psychological variables.

**Results:**

This study was funded in January 2023. Data collection for baseline assessments began in February 2023. Formal data analysis started in April 2025 after the 6-month follow-up assessments were completed.

**Conclusions:**

To the best of our knowledge, this study will be the first randomized controlled trial to combine T2D genetic risk communication with wearable device functions in any population. Novel findings will be used to inform future lifestyle modification strategies for T2D. We plan to provide a comprehensive report on this study by publishing this analysis plan before the completion of data collection.

**Trial Registration:**

ClinicalTrials.gov NCT05524909; https://www.clinicaltrials.gov/study/NCT05524909

**International Registered Report Identifier (IRRID):**

DERR1-10.2196/65012

## Introduction

### Trial Background and Rationale

Currently, approximately 500 million individuals worldwide live with diabetes, of whom 90% have type 2 diabetes (T2D) [1]. By 2045, the number of patients with diabetes is projected to reach 170 million in China alone [2,3]. Therefore, developing strategies to address the increasing prevalence of T2D cases remains a key public health priority across East Asian countries.

Compared to White populations, East Asian populations have a higher risk for diabetes despite having lower mean BMI levels. An early study conducted among Hong Kong Chinese adults demonstrated that diabetes risk increased at a BMI of approximately 23 kg/m^2^, which did not conform to BMI cutoff values applied to White populations [4]. Subsequent findings from 2 Asian American cohort studies further supported the notion that individuals of East Asian descent have an elevated risk for T2D, as they have greater adiposity than their White counterparts within each BMI category [5,6]. Epidemiological evidence from Hong Kong also revealed that individuals aged between 40 and 60 years accounted for 40% of incident T2D cases [7] and that increased hazard ratios for cardiovascular diseases, cancer, and all-cause mortality were observed among middle-aged adults with prediabetes [8]. Therefore, it is crucial to develop interventions specifically targeting East Asian middle-aged adults with overweight or obesity, given their higher susceptibility to T2D.

Being overweight or obese is a major risk factor for T2D and is typically characterized by prolonged sedentary time and chronic physical inactivity. New exercise guidelines have since been established to promote reductions in sedentary behavior and increases in moderate to vigorous physical activity (MVPA) [9]. Evidence from recent observational and isotemporal substitution studies has demonstrated the benefits of increased accelerometer-derived MVPA time on T2D, ranging from lowered incidence of T2D-related vascular complications to improved insulin sensitivity and glycemic control [10-13]. Taken together, these findings highlight the key role of MVPA in improving health among individuals with overweight or obesity who are at risk for T2D.

The defining characteristic of T2D is the decline in pancreatic beta cell functions, caused primarily by chronic insulin resistance. Although research has uncovered a plethora of risk factors implicated in T2D progression, such as age, diet, physical inactivity, alcohol consumption, BMI, and family history [14], the multifactorial nature of T2D makes it challenging for any single approach to accurately screen for and predict disease risk among individuals at risk.

Owing to advances in genomics and precision medicine, technologies for assessing genetic predisposition to noncommunicable diseases have become increasingly accessible. Coupled with recent genome-wide association studies, these advances have enabled the discovery of genetic variants that exhibit significant associations with T2D among East Asian populations. Accurate genomic sequencing can facilitate genetic risk communication for T2D on an unprecedented scale. This could provide researchers with potential insights into the impact of T2D genetic risk communication on behavioral changes among East Asians.

However, evidence from multiple studies has indicated that communication of genetic risk has limited impact on behavioral changes [15], raising concerns regarding the overall effectiveness of this approach. Nonetheless, these studies share several limitations: (1) genetic risk was communicated without considering behavioral change theories; (2) MVPA, when used as a primary outcome, was often measured through self-report-based measures; (3) genetic risk for polygenic diseases was calculated using singular genetic variants; and (4) most of the studies focused on individuals of European descent, limiting the generalizability of the findings to East Asian populations.

Considering the steady growth of the wearable device market, consumer-based physical activity (PA) trackers have shown promise in serving as a low-cost platform for intervention studies.

Contemporary wearable devices often feature streamlined interfaces and burnished designs and encourage PA behaviors through goal setting and self-monitoring. For researchers, wearables provide objective measurements of lifestyle behaviors while retaining mass appeal for smooth implementation. Previous randomized controlled trials and systematic reviews have shown that interventions incorporating a goal-setting function (eg, at least 10,000 steps per day) in wearables showed high efficacy in decreasing BMI and supporting long-term weight management among participants with overweight or obesity [16-18]. A recent meta-analysis reported that using wearables as a key intervention modality led to an average increase of 1800 daily step counts, 40 minutes of walking time, and 6 minutes of MVPA time [19]. However, communication of genetic information was not included in the intervention, and the interplay between wearable device functions and T2D genetic risk communication remains unclear.

Herein, we present the detailed data analysis plan for, to the best of our knowledge, the first East Asian–focused randomized controlled trial using wearable device functions in conjunction with genetic risk communication to facilitate changes in MVPA. The wearable device (Fitbit Inspire 3) used in the trial includes a built-in accelerometer, enabling the collection of objectively measured PA data. Fitbit functions such as activity goal setting and activity prompts will play a central role in promoting and sustaining MVPA. By combining personalized precision medicine with wearable device functions, the trial aims to provide novel insights into lifestyle modification strategies.

### Trial Objectives and Hypothesis

The main objectives of this trial are to (1) investigate the impact of T2D genetic risk communication and (2) examine the effects of combining T2D genetic risk communication with theory-based wearable device functions (eg, step goal setting and activity prompts commonly found in consumer-grade wearables) on wearable device–measured MVPA time among East Asian middle-aged adults with overweight or obesity.

We hypothesize that T2D genetic risk communication alone will result in minimal increases in objectively measured MVPA time, whereas combining T2D genetic risk communication with wearable device functions will significantly increase MVPA time not only at the 12-month postintervention time point but also at the 6-month follow-up.

## Methods

### Recruitment

Participants were recruited across various communities in Hong Kong primarily via flyer distribution, local minibus advertisements, emails, and word of mouth. Recruitment materials included the study title, research question, brief study information (eg, study period, duration, and key particulars), participant incentives, inclusion and exclusion criteria, sign-up methods, and contact information for the research team.

Flyers were distributed alongside pull-up banners within local communities, and minibus advertisements were placed on the back of local minibus seats. Recruitment emails were circulated within local organizations and community centers. In brief, flyer distribution (183/355, 51.5%) and minibus advertisements (142/355, 40%) accounted for most of the recruited participants, while emails (19/355, 5.4%) and word of mouth (11/355, 3.1%) accounted for the remainder. Interested individuals were asked to complete an online screening form when signing up.

### Trial Design

#### Overview

**Figure 1:**
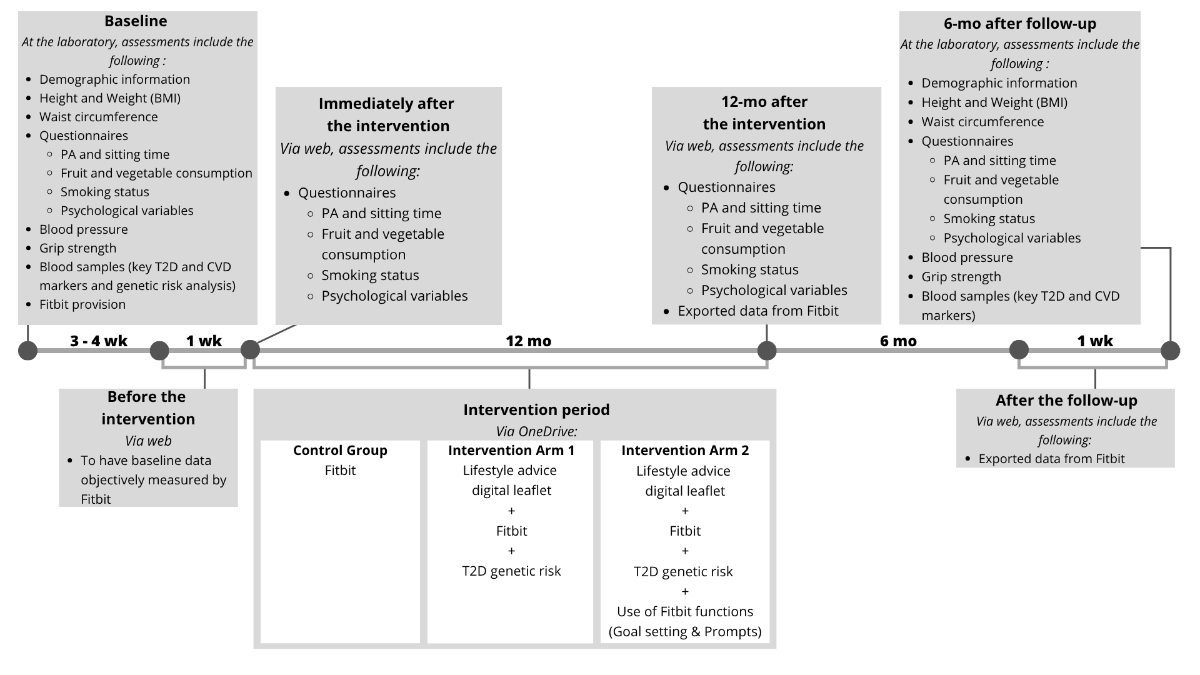
Overall study timeline and protocol for genetic risk communication and wearable device interventions. CVD: cardiovascular disease; PA: physical activity; T2D: type 2 diabetes.

Figure 1 shows the timeline and protocol of the study. This parallel-group randomized controlled trial commenced on May 11, 2023, and aims to involve 355 participants. Each participant is randomly allocated to 1 of 3 groups: 1 control group and 2 intervention groups (intervention arm 1 and intervention arm 2). The inclusion of 2 intervention arms allows examination of the effects of T2D genetic risk communication alone as well as the combined effects of T2D genetic risk communication and wearable device functions. All participants are asked to wear a Fitbit device throughout the study period for measuring outcome data.

Participants in intervention arm 1 receive a Fitbit device, T2D genetic risk estimates, and a digital lifestyle leaflet. Participants in intervention arm 2 also receive these materials and have their Fitbit devices calibrated such that their daily step goal is 10% higher than their baseline step count. In addition, they receive activity prompts through the Fitbit device. The control group receives only a Fitbit device. The daily step goals for participants in intervention arm 2 are set 10% above baseline based on relative efficiency and higher feasibility. Chapman et al [20] reported that implementing a 50% increase in baseline step count did not result in greater walking activity compared to a 10% increase. Moreover, only 5% of participants did not meet their step goals when given a 10% step goal increase over baseline compared to 16% who were given a 50% increase [20]. On the basis of these findings, implementing a 10% increase over baseline was considered more feasible and effective for motivating participant behavior.

This trial has been registered at ClinicalTrials.gov (NCT05524909).

#### Inclusion Criteria

Eligibility criteria for participants were (1) being aged between 40 and 60 years, (2) being of East Asian ancestry, (3) having a BMI ≥23 kg/m^2^ (according to the World Health Organization BMI cutoffs for Asians), (4) being able to comprehend English or Chinese, and (5) owning a smartphone.

#### Exclusion Criteria

Participants were excluded if they (1) were unable to perform routine daily PAs (as determined by the Physical Activity Readiness Questionnaire), (2) had prior experience with consumer-directed genetic testing, (3) had been diagnosed with any type of diabetes, or (4) were participating in any exercise-related intervention study.

### Intervention

T2D genetic risk estimates, together with a digital lifestyle leaflet, are delivered weekly via WhatsApp (Meta) and monthly via email, as results from prior studies suggest that genetic risk information can promote optimal health behavior changes when communicated at regular and repeated intervals [21,22]. A total of 113 single nucleotide polymorphisms (SNPs), with corresponding odds ratios and allele frequency values for T2D genetic risk, were derived from a genome-wide association study that identified SNPs associated with T2D risk among East Asians [23]. The estimation procedure has been illustrated in our published protocol and supplementary materials of our previous report [24,25]. Participants would then receive their estimated 10-year and remaining lifetime genetic risk for developing T2D, as well as a dichotomized genetic risk category in comparison to the average population risk for T2D (according to the participant’s age and sex): “increased genetic risk” and “no increased genetic risk.” The digital leaflet contains detailed information on the definition and health impacts of T2D, as well as lifestyle advice on 4 major T2D risk factors (ie, PA, diet, weight management, and smoking) as recommended by the World Health Organization.

Participants in intervention arm 2 receive two unique Fitbit features—(1) step goal setting and (2) activity prompts—in addition to the T2D genetic risk estimates and digital leaflet. Each participant’s step goal is set by staff to be 10% higher than their baseline step count, calculated as the average of the 7-day preintervention Fitbit data. The “Reminder to move” function is used as a prompt to interrupt prolonged sedentary periods. When the wearer has not accumulated more than 250 steps within an hour, the device vibrates and displays a reminder message. We have specified an operation period for this function, and the device reminds participants 10 minutes before each hour between 9 AM and 10 PM. All Fitbit functions unique to this intervention can be accessed and modified only by researchers via the Fitbit online dashboard system.

### Randomization

A block randomization approach has been adopted. The randomization list was generated using a computer program by a staff member who had no prior knowledge of participant information. Each block consisted of 6 allocations, with 2 participants assigned to each of the 3 groups per block.

Group allocation has been concealed from study staff until the 7-day preintervention period, during which staff would have prepared the corresponding intervention materials for participants. The participants will be initially blinded to group assignment; however, blinding will not be possible once the intervention is delivered. A statistician blinded to group assignment will perform outcome analyses using deidentified data.

### Sample Size

To ensure sufficient statistical power, we intend to recruit 355 East Asian participants with overweight or obesity. An oversampling strategy has been adopted, with 35.1% (87/248) of the full sample consisting of individuals at a higher genetic risk for T2D. The required number of participants (n=87) for subsample analysis was based on the desired medium effect size (*F*=0.3085), derived from a 3-group comparison. The control group’s average wearable device–measured MVPA time was 80 (SD 35) min/d. The intervention group receiving genetic risk information alone (intervention arm 1) demonstrated minimal changes (85 min/d), while the intervention group receiving combined genetic risk information and wearable device functions (intervention arm 2) was expected to show a difference of approximately 23% in MVPA time (105 min/d; [(105 − 85) / 85 × 100%], corresponding to a difference of 23% in MVPA time compared to previous Fitbit interventions) [26].

Our sample size calculation assumed an α level of 5%, a power of 80%, and a correlation of 0.70 among 3 repeated measures. A total of 355 participants are recruited to ensure the inclusion of an analysis sample of 248, while factoring in an expected attrition rate of 20% (71/355) and an anticipated Fitbit data missing rate of 10.1% (36/355), independent of attrition, considering instances of insufficient battery life or device loss.

### Framework

We adopt a superiority hypothesis testing framework for this trial, examining whether combining T2D genetic risk communication with theory-based wearable device functions will lead to significant increases in objectively measured MVPA time and whether such changes will be sustained at the 6-month postintervention follow-up. Each intervention group (intervention arm 1 and intervention arm 2) will be compared separately with the control group and with each other.

### Interim Analysis and Stopping Guidance

No interim analysis is planned for this study, as the intervention is not expected to cause harm to participants or raise safety concerns that would warrant ending the trial prematurely.

### Ethical Considerations

This study was reviewed and approved by the institutional review board of the University of Hong Kong (HKU) and Hospital Authority Hong Kong West Cluster on December 8, 2022 (UW 22-007).

A subject information sheet and a written informed consent form were provided to each study participant before any assessment or data collection. Participants were given sufficient time to read through both documents and the opportunity to ask questions. The consent form included a section asking whether participants agreed to allow the data from this study to be used in future ancillary research. Participants were informed that they could withdraw from the study at any stage without providing a reason.

All study materials and records are stored securely at the study site. All electronic participant records and responses are maintained on the network drives of the HKU research laboratory. Hard copies of study materials are stored in locked filing cabinets at the HKU research laboratory. Only project staff have access to the study materials and data. All study materials and data will be shredded or otherwise destroyed 5 years after study completion. Deidentified data will be used in outcome data analysis by a study staff member who is blinded to participant group assignment.

Upon study completion, participants will retain their Fitbit Inspire 3 device as an incentive. All Fitbit devices were purchased from a local retail supplier in Hong Kong, with costs supported by the General Research Fund provided by the Hong Kong Research Grants Council (grant 17115422).

### Outcomes

#### Primary Outcome

MVPA, defined as the sum of “fairly active minutes” and “very active minutes” measured by the Fitbit device, is the primary outcome of this study. MVPA will be measured at baseline, immediately after the intervention, 12 months after the intervention, and at the 6-month follow-up.

#### Secondary Outcomes

All measurements of secondary outcomes will be conducted at baseline, immediately after the intervention, 12 months after the intervention, and at the 6-month follow-up. The secondary outcomes are presented in Textbox 1.

**Secondary outcomes.**

**Laboratory assessment variables**

- BMI (kg/m^2^; derived from height and weight measurements)
- Systolic and diastolic blood pressure (mm Hg; measured using the Omron HEM-907 digital automatic blood pressure monitor)
- Resting heart rate (beats per minute; measured using the Omron HEM-907 digital automatic blood pressure monitor)
- Hand grip strength (kg; measured using the Jamar Hydraulic Hand Dynamometer [Fabrication Enterprises])

**Blood-based metabolic risk biomarkers**

- Glycated hemoglobin (%)
- Total cholesterol (mmol/L)
- High-density lipoproteins (mmol/L)
- Low-density lipoproteins (mmol/L)
- Triglycerides (mmol/L)

Fitbit device variables

- Steps
- “Sedentary minutes” (minutes)
- “Lightly active minutes” (minutes)
- “Fairly active minutes” (minutes)
- “very active minutes” (minutes)
- “Activity calories” (kcal)
- Sleep time (minutes)

**Self-reported variables (assessed using a series of questionnaires)**

- Physical activity and sitting time
- Fruit and vegetable consumption
- Smoking status
- Psychological variables to determine the effects of genetic risk communication

### Timing of Outcome Assessments

The time points for outcome measurements during the trial are presented in Table 1.

**Table 1:** Schedule of assessments.

Assessments	Time points				
	Baseline	Immediately after the intervention	12 months after the intervention	6-month follow-up	After the follow-up
MVPA^a^ time (minutes)	✓	✓	✓		✓
Steps	✓	✓	✓		✓
Sedentary time (minutes)	✓	✓	✓		✓
Sleep time (minutes)	✓	✓	✓		✓
Lightly active time (minutes)	✓	✓	✓		✓
Fairly active time (minutes)	✓	✓	✓		✓
Very active time (minutes)	✓	✓	✓		✓
Activity calories (kcal)	✓	✓	✓		✓
Psychological variables of genetic risk communication	✓	✓	✓	✓	
PA^b^ and sitting time	✓	✓	✓	✓	
Fruit and vegetable consumption	✓	✓	✓	✓	
Smoking status	✓	✓	✓	✓	
Blood-based metabolic risk biomarkers	✓			✓	
Height (cm)	✓			✓	
Weight (kg)	✓			✓	
Handgrip strength (kg)	✓			✓	
Systolic and diastolic blood pressure (mm Hg)	✓			✓	
Resting heart rate (BPM^c^)	✓			✓	

^a^MVPA: moderate to vigorous physical activity.

^b^PA: physical activity.

^c^BPM: beats per minute.

### Timing of Final Statistical Analyses

All planned statistical analyses will be performed after the completion of the trial.

### Statistical Principles

We will present 95% CIs with adjusted marginal means to compare differences between groups at each measurement time point (baseline, immediately after the intervention, 12 months after the intervention, and 6-month follow-up). Results for the interaction term will also be presented. Pairwise between-group comparisons will be performed with Bonferroni adjustment. An overall level of statistical significance will be set at α=.05 before adjustment.

### Adherence and Protocol Deviations

This study allows researchers a high level of control over protocol adherence and deviations. Given that intervention materials are mainly delivered weekly via WhatsApp messages, and only researchers have full control over Fitbit wearable functions, the potential for protocol deviation is minimal. Research staff will perform Fitbit data consistency checks via the Fitbit online dashboard system, which includes (1) monitoring battery life and synchronization status and (2) downloading Fitbit data. Reminders to charge and synchronize the device will be sent via WhatsApp if participants have not done so. Participants are welcome to approach staff should any issues arise. Participants are told to feel free to request withdrawal from the study at any stage.

To investigate the effects of adherence and device wear time, a post hoc analysis will compare participants with consistent wear time to those with inconsistent wear time across all groups.

### Analysis Populations

Primary outcome analysis will be conducted according to intention-to-treat principles. Participants with missing outcome data at the 6-month follow-up will be excluded from the analysis.

### Trial Population

#### Screening Data

No screening data are collected. Participants’ eligibility is assessed on site.

#### Recruitment

We will report the following: (1) the number of participants eligible for baseline assessment, (2) the number of participants randomly assigned to each group, (3) the number of participants who completed the 6-month follow-up, and (4) the number of participants included in the main analysis (recording any exclusion with reasons).

#### Withdrawal and Loss to Follow-Up

Participants are free to request withdrawal from the study at any stage without providing a reason. The number of participants who withdraw due to reasons such as serious adverse events, safety concerns, or the development of health conditions or symptoms is reported for each randomly assigned group at all outcome measurement time points.

#### Baseline Characteristics

The following baseline characteristics will be recorded and summarized for the full sample and by trial arm: (1) age; (2) sex; (3) educational level; (4) body weight and height; (5) BMI; (6) nondominant hand; (7) blood pressure (systolic and diastolic); (8) handgrip strength (both left and right hands); (9) estimated 10-year and remaining lifetime genetic risk for developing T2D; and (10) blood-based metabolic risk biomarkers such as total cholesterol, glycated hemoglobin, high-density lipoproteins, low-density lipoproteins, and triglycerides.

For categorical variables, we will present the proportion and total number of participants within each category. For continuous variables, means and SDs will be presented. If data are skewed, medians and IQRs will be presented instead.

### Analysis Methods

#### Primary Outcome

We will perform primary outcome analysis according to intention-to-treat principles using a series of linear mixed effects models with fixed effects for time, group, and group × time interactions for objectively measured MVPA time, with repeated measures for each participant. Models will be adjusted for potential confounders such as age, sex, and BMI. Adjusted marginal means with 95% CIs will also be presented to compare differences between study groups across all assessment time points.

The analysis will use a longitudinal data analysis model to examine patterns of differences across assessment time points, study groups, and group × time interactions. Results for the group × time interaction term will be included. In addition to the 4 assessment time points, the analysis will also be performed with time as a continuous variable across the study period. The effect modification will also be investigated according to the level of T2D genetic risk, which will be further categorized based on the average T2D genetic risk estimate and educational level of the sample (ie, between college and noncollege graduates). This post hoc categorization can enable us to examine the intervention effects within each group of genetic risk. To ensure fair random assignment of participants between the groups, we will examine between-group differences in the primary outcome data, using an ANOVA test or, if parametric assumptions are not met, a Kruskal-Wallis test.

#### Secondary Outcomes

Analysis of continuous secondary outcomes will be conducted in a manner similar to that used for the primary outcome. For continuous variables such as blood-based metabolic risk biomarker variables, additional Fitbit device–measured variables, and laboratory assessment variables, we will perform an ANOVA test or, if parametric assumptions are not met, a Kruskal-Wallis test. To analyze differences in categorical variables, such as smoking status and psychological variables related to T2D genetic risk communication, we will conduct chi-square tests.

#### Sensitivity Analysis and Missing Data

We will conduct two sets of sensitivity analyses: (1) without adjustment for confounders and (2) using an iterative Markov chain Monte Carlo multiple imputation procedure to handle missing data at the 12-month postintervention and 6-month follow-up time points. This procedure assumes that data are missing at random within each group. The randomness of missing data will be tested using the *Amelia* package in R. For variables with missing values (eg, sex, age, educational level, BMI, blood pressure, and handgrip strength at baseline), 5 imputations will be generated for each missing value. To account for nonlinear trajectories in PA patterns, we will conduct additional sensitivity analyses using nonlinear mixed effects models with spline terms to capture nonlinear trajectories in outcome variables.

#### Safety Considerations

This is a low-risk trial and is not expected to have any major adverse effects on participants’ health. The Physical Activity Readiness Questionnaire plays a key role in identifying risk factors such as cardiovascular conditions and musculoskeletal problems. Research staff can assess participants’ health status and reduce the likelihood of injuries and other adverse events. As no adverse events are expected from participating in any of the interventions, formal monitoring of adverse events will not be conducted. However, participants may exhibit some degree of discomfort, distress, or psychological concerns regarding their genetic risk for T2D. To address this potential issue, we aim to assess participants’ psychological responses to genetic risk communication via web-based questionnaires, which will be administered at all 4 measurement time points. Participants in the intervention groups may also misinterpret genetic risk estimates. To mitigate this, representation of diabetes risk will be assessed using the Brief Illness Perceptions Questionnaire, which consists of 9 items designed to assess cognitive and emotional representations of illness. More detailed descriptions can be found in Multimedia Appendix 1 [25,27-36]. All participants are informed of the full scope and content of the study before participation and are told that they may withdraw from the study at any time without providing a reason.

## Results

This study was funded in January 2023, and baseline data collection began in February 2023. The recruitment of 355 participants was completed in August 2023. As of February 2025, the study had entered the 6-month follow-up stage. Formal data analysis will start in April 2025 after the 6-month follow-up assessments have been completed. We expect the results to be published in December 2025.

## Discussion

### Summary

We hypothesize that intervention arm 1 will show minimal increases in objectively measured MVPA time, whereas intervention arm 2 will demonstrate significantly increased MVPA time not only at the 12-month postintervention time point but also at the 6-month follow-up.

### Strengths and Limitations

This study has notable strengths. It provides a multicomponent intervention combining personalized T2D genetic risk estimates with wearable device functions. Previous trials have investigated the effects of genetic risk communication, but none used behavioral theory–based wearable device functions in conjunction with genetic risk communication [37,38]. Moreover, our study calculates genetic risk estimates based on 113 SNPs, which allows for more precise estimation of T2D genetic risk compared to studies that included only a few SNPs [39,40].

There are also limitations. As residential locations of participants are not collected, and they were instructed to carry out their regular PA routines, it is possible that neighborhood environments could skew measurement outcomes by modulating intervention effects [41,42]. The control group receives a Fitbit device without additional intervention, which could introduce Hawthorne effects, as participants may alter their behavior due to awareness of being studied. To mitigate this, only research staff have full control over the additional Fitbit functions, ensuring that participants in intervention arm 2 use the Fitbit functions pertaining to the intervention (ie, step goal setting and activity prompts). Regular Fitbit data consistency checks are also performed to prevent participants in the control group from accessing these functions. Finally, previous studies have highlighted the short-term nature of Hawthorne effects [43,44]. As measurement of outcome data is being conducted over a period of 7 days at each time point, any impact of Hawthorne effects is likely to be minimized.

### Conclusions

This study will be the first randomized controlled trial to combine T2D genetic risk communication with wearable device functions in any population. Novel findings will be used to inform future lifestyle modification strategies for T2D. We plan to provide a comprehensive report on this study by publishing this analysis plan before the completion of data collection.

## Appendix

Multimedia Appendix 1 Measures used in the assessment questionnaire.

Multimedia Appendix 2 CONSORT-eHEALTH checklist (V 1.6.1).

Multimedia Appendix 3 Peer-review report from the General Research Fund and Early Career Scheme, Hong Kong Research Grants Council.

## Abbreviations

HKU: University of Hong Kong
MVPA: moderate to vigorous physical activity
PA: physical activity
SNP: single nucleotide polymorphism
T2D: type 2 diabetes

## Data Availability

The datasets generated or analyzed during this study are not publicly available but are available from the corresponding author on reasonable request.
