# Supplementary material for: Effects of Communicating Genetic Risk of Type 2 Diabetes and Wearable Technologies on Behavioral Outcomes in East Asians: Statistical Analysis Protocol for a Randomized Controlled Trial
Source: JMIR Res Protoc. 2025 Nov 5;14:e65012. doi: 10.2196/65012 (PMC12631090; doi:10.2196/65012)
Supplement: Multimedia Appendix 1 [file resprot_v14i1e65012_app1.docx]

**Measures used in the assessment questionnaire**

| Anxiety | Assessed using the 6-item short-form of the state scale of the Spielberger State-Trait Anxiety Inventory (STAI) [a]. Results obtained from this short-form STAI have shown to be comparable to those obtained using the full-form of the STAI. It has been used in our previous published research [b] to measure anxiety related to diabetes risk communication. |
| --- | --- |
| Perception of fruit and vegetable consumption | Assessed using 2 items developed by our previous research [b] to assess anxiety related to diabetes risk communication. Participants are asked 1) how much fruit and vegetables they think that they eat compared to people of their age and sex, and answer on a 5-point response scale, ranging from “much less” to “much more”; 2) whether or not they meet the WHO and local recommendations for fruit and vegetable consumption. |
| Perception of physical activity | Assessed using 2 items developed by our previous published [b] research to measure anxiety related to diabetes risk communication. Participants are asked 1) how physically active they think that they are compared to people of their age and sex, and answer on a 5-point response scale, ranging from “much less” to “much more”; 2) whether or not they meet the WHO and local recommendations for engagement in physical activity. |
| Physical activity | Assessed using International Physical Activity Questionnaire Short Form (IPAQ-SF) which has been shown to produce reliable responses that are reflective to objectively measured physical activity [c]. |
| Smoking status | Assessed using the 3 basic items recommended in the Tobacco Questions for Surveys - A Subset of Key Questions from the Global Adult Tobacco Survey (GATS) 2nd Edition [d]. In addition, an item assessing the number of cigarettes smokers use daily is included. |
| Intentions to be physically active, consume adequate fruit and vegetables, and avoid smoking | Assessed using 3 items for each health behavior. These items have been adapted according to the recommendations provided by Ajzen [e] and our previous published research [f]. Each item includes a statement (e.g. “I intend to meet the recommendations for physical activity over the next 4 weeks.”) evaluated on a 7-point Likert scale. |
| Perceived risk | Assessed using 6 items. Participants are asked 1) how likely they think that they are to get T2D in the next 10 years and their lifetime, and first answer on a 5-point response scale, ranging from “very unlikely” to “very likely”, and then on a continuous scale, ranging from 1 to 100; 2) how likely they think they are to get T2D in the next 10 years and their lifetime, compared to people their same age and sex, and answer on a 5-point response scale, ranging from “much less likely” to “much more likely”. These items have been used in previous research [b,g]. |
| Diabetes-related worry | Assessed using the six-item Cancer Worry Scale (CWS) [h] which identifies dimensions that are relevant in the genetic counselling context, such as worry about developing breast cancer, impact of worries on daily life, and risk perception. These items have been adapted for use in the context of T2D in our previous research [b] and have been shown to maintain its good construct validity, convergent and divergent validity, and high internal consistency in recent years [i]. |
| Diabetes risk representations | Assessed using the Brief Illness Perceptions Questionnaire (Brief IPQ) [j] which originally consists of 9 items designed to assess the cognitive and emotional representations of illness. It has shown good test–retest reliability and concurrent validity with relevant measures.83 To measure T2D risk representations held by healthy individuals, items 1-8 of Brief IPQ have been adapted and the last item has been excluded according to methods used in previous research [b,k]. In addition, 12 items adapted from Revised Illness Perception Questionnaire (IPQ-R) assessing perceived severity and causal attributions are included according methods used in previous research [b,k]. |
| Self-efficacy | Assessed using 3 items for each health behavior. These items have been adapted according to the recommendations provided by Ajzen [e] and our previous published research [f]. Each item includes a statement (e.g. “It is easy for me to meet the recommendations for physical activity over the next 4 weeks.”) evaluated on a 7-point Likert scale. |

1. Marteau TM, Bekker H. The development of a six-item short-form of the state scale of the Spielberger State-Trait Anxiety Inventory (STAI). 1992.
2. Godino JG, van Sluijs EMF, Marteau TM, Sutton S, Sharp SJ, Griffin SJ. Effect of communicating genetic and phenotypic risk for type 2 diabetes in combination with lifestyle advice on objectively measured physical activity: protocol of a randomised controlled trial. BMC Public Health 2012, 12(1):444-444.
3. Ekelund U, Sepp H, Brage S, Becker W, Jakes R, Hennings M, Wareham NJ. Criterion-related validity of the last 7-day, short form of the International Physical Activity Questionnaire in Swedish adults. Public Health Nutr 2006, 9(2):258-265.
4. Global Adult Tobacco Survey Collaborative Group. Tobacco Questions for Surveys: A Subset of Key Questions from the Global Adult Tobacco Survey (GATS), 2nd Edition, Atlanta, GA: Centers for Disease Control and Prevention, 2011.
5. Ajzen I. Constructing a TpB Questionnaire: Conceptual and Methodological Considerations. In: 2002; 2002.
6. Chan DKC, Stenling A, Yusainy C, Hikmiah Z, Ivarsson A, Hagger MS, Rhodes RE, Beauchamp MR. Editor's Choice: Consistency tendency and the theory of planned behavior: a randomized controlled crossover trial in a physical activity context. Psychol Health 2020, 35(6):665-684.
7. Isaac ML, Maggie K, Colleen MM, Hayden BB, Kathryn IP, Ilene CS, Barbara KR. Relationships among Breast Cancer Perceived Absolute Risk, Comparative Risk, and Worries. Cancer epidemiology, biomarkers & prevention : a publication of the American Association for Cancer Research, cosponsored by the American Society of Preventive Oncology 2000, 9(9):973.
8. Caruso A, Vigna C, Gremigni P. The Cancer Worry Scale Revised for Breast Cancer Genetic Counseling. Cancer Nurs 2018, 41(4):311-319.
9. Custers JAE, Kwakkenbos L, van de Wal M, Prins JB, Thewes B. Re-validation and screening capacity of the 6-item version of the Cancer Worry Scale. Psychooncology 2018, 27(11):2609-2615.
10. Broadbent E, Petrie KJ, Main J, Weinman J. The Brief Illness Perception Questionnaire. J Psychosom Res 2006, 60(6):631-637.
11. Figueiras MJ, Alves NC. Lay perceptions of serious illnesses: An adapted version of the Revised Illness Perception Questionnaire (IPQ-R) for healthy people. Psychology & health 2007, 22(2):143-158.
