# Supplementary material for: Effects of Communicating Genetic Risk of Type 2 Diabetes and Wearable Technologies on Behavioral Outcomes in East Asians: Statistical Analysis Protocol for a Randomized Controlled Trial
Source: JMIR Res Protoc. 2025 Nov 5;14:e65012. doi: 10.2196/65012 (PMC12631090; doi:10.2196/65012)
Supplement: Multimedia Appendix 3 [file resprot_v14i1e65012_app3.pdf]

VIEW COMMENTS FROM PANEL

**Project Number :**	17115422
**Project Title :**	Combined Effects of Communicating Genetic Risk of Type 2 Diabetes and Wearable Technologies On Objectively Measured Behavioral Outcomes in Overweight or Obese East Asian Individuals.
**PI Name :**	Dr Kim Youngwon

Overall Comments:
It addresses a major public health challenge (childhood obesity) with newly, and specifically formulated hypotheses: the effects of communicating genetic risk for type 2 diabetes (T2D) and a wearable device. There are minor concerns that can be addressed with reasonable efforts.

Strengths:
Both hypotheses are important for mitigating overweight risks, and the increased sample size and updated design addressed the concerns raised in the previous review.

Weaknesses:
The formulation of hypotheses seem to be 3: (1) “communication” of genetic risk ALONE is effective, (2) “utilization” of step-goal setting and prompt functions ALONE is effective, (3) combination of both is even MORE effective. If so, four groups (plus the control group with neither measure), instead of currently designed three groups, would need to be planned.
Additional measures may be employed to ensure wide representation of samples from diverse socioeconomic groups and wide geographic areas.

No new comments

Potential Research Impact Comment:

It is a very important project with direct implications on public health policy.

---

high impact

Research Grants Council 研究資助局

General Research Fund and Early Career Scheme

VIEW COMMENTS FROM EXTERNAL REVIEWER 1

Project Number : 17115422

Project Title : Combined Effects of Communicating Genetic Risk of Type 2 Diabetes and Wearable Technologies On Objectively Measured Behavioral Outcomes in Overweight or Obese East Asian Individuals.

PI Name : Dr Kim, Youngwon

Section A : Detailed Comments

1. Please comment on the objective(s) of the proposal, and whether the research agenda adequately addresses the objective(s)?

Excellent	Very Good	Good	Fair	Poor
○	●	○	○	○

Comments:

Objectives are adequate to meet the criteria of research agenda

2. Please comment on the Research Design and Methodology.

Excellent	Very Good	Good	Fair	Poor
○	○	●	○	○

Comments:

Methodology did not address the interventions strategies and tools that would be used as communication activities

3. Please comment on the feasibility of the proposed research.

Excellent	Very Good	Good	Fair	Poor
○	○	○	●	○

Comments:

justification for the proposed duration should be explained

4. What do your consider to be the most original or innovative aspect of the proposed research? What advances would the research result bring about to the related field if the proposed research is successful?

Comments:

the proposal needs to address communication tools and theoretical framework. the basic concepts are good

5. Please comment on the reasonableness of the proposed budget and manpower planning and project duration.

Comments:

budget is reasonable. it may demand more as intervention period extends for measuring impact

6. Overall Comments

Overall Comment : the proposal has a practical value. risk communication is important component for the intervention

Strength: identification of target population, communication intervention for behavior chance

y
Weaknesses: analysis on communication intervention should be presented adequately. justification of behavior change should be added

Suggested improvements: needs detailed explanation on implementation of communication activities. justification for behavior change model must be reflected in the communication intervention activities

Section B : Summary of Assessment

The project :

**Scientific/scholarly merit**	Excellent	Very Good	Good	Fair	Poor
	○	●	○	○	○
**Duration Proposed**	Too Long	Appropriate	Too Short		
	○	○	●		
**Impact of Research**	High	Moderate	Low	None	
	●	○	○	○	

The principal investigator :

	Excellent	Very Good	Good	Fair	Poor
Ability to undertake the proposal	○	◉	○	○	○
Track record in field	○	◉	○	○	○

VIEW COMMENTS FROM EXTERNAL REVIEWER 2

Project Number : 17115422
Project Title : Combined Effects of Communicating Genetic Risk of Type 2 Diabetes and Wearable Technologies On Objectively Measured Behavioral Outcomes in Overweight or Obese East Asian Individuals.
PI Name : Dr Kim, Youngwon

Section A : Detailed Comments

1. Please comment on the objective(s) of the proposal, and whether the research agenda adequately addresses the objective(s)?

Excellent	Very Good	Good	Fair	Poor
○	○	◉	○	○

Comments:

The proposed project has two objectives: 1) determine the effects of communicating genetic risk for type 2 diabetes (T2D), and the hypothesis here is that such communication by itself would have little effect on motivating the physical activity; and 2) determining the effect of combining the communication of genetic risk and utilization of step-goal setting and prompt functions of a wearable device, and the hypothesis here is that this combination is much more effective than solely using the communication of the genetic risk. The research design for achieving these determinations is a case-control study that splits random sample subjects into 3 groups that receive different interventions and compares the outcomes of the groups. Generally, the design addresses the objectives.

2. Please comment on the Research Design and Methodology.

Excellent	Very Good	Good	Fair	Poor
○	○	◉	○	○

Comments:

Maybe I have missed something, but I sense that the three-group case-control study has a flaw that would make the comparison not able to achieve the targeted conclusion. The two objectives of the project are essentially targeting a single hypothesis: either "communication" of genetic risk OR "utilization" of step-goal setting and prompt functions would NOT achieve the most optimal effect; instead, the "combination" of them will. The current design, however, logically can only determine if the "communication" by itself is effective or not, but cannot determine if the "utilization" by itself is effective or not, and therefore cannot ultimately determine if the "combination" is better than either.

Also, the proposal did not really explain the reason for setting the step goals 10% higher than the baseline average step counts, but just refer to a reference, which is not helpful to justifying the logic underlying this setting and more importantly, the validity of this setting in the comparison of different groups.

3. Please comment on the feasibility of the proposed research.

Excellent	Very Good	Good	Fair	Poor
○	◉	○	○	○

Comments:

The proposed research is feasible.

4. What do your consider to be the most original or innovative aspect of the proposed research? What advances would the research result bring about to the related field if the proposed research is successful?

Comments:

The hypothesis that a combination of communication of certain information and utilization of certain physical reminders will achieve more optimal efficacy than solely applying either of them is original and innovative. If the project is successful, it is seminal and inspiring to similar studies and will have important implications in designing clinical interventions.

5. Please comment on the reasonableness of the proposed budget and manpower planning and project duration.

Comments:

The proposed budget and manpower, as well duration are reasonable.

6. Overall Comments

Overall Comment: The project has a potential to succeed, but the design needs further consideration.

Strength: The idea is novel. The research design is generally good. The statistical analysis is thoughtful.

Weaknesses: There seems to be a logical flaw in the case-control study that needs to be fixed.

Suggested improvements: Either modify the case-control study to address the flaw described above or clearly explain why it is not a flaw.

Section B : Summary of Assessment

The project :

	Excellent	Very Good	Good	Fair	Poor
**Scientific/scholarly merit**	○	◉	○	○	○

**Duration Proposed**	**Too Long**	**Appropriate**	**Too Short**		
	○	◉	○		
**Impact of Research**	**High**	**Moderate**	**Low**	**None**	
	○	◉	○	○	

The principal investigator :

**Ability to undertake the proposal**	**Excellent**	**Very Good**	**Good**	**Fair**	**Poor**
	◉	○	○	○	○
**Track record in field**	**Excellent**	**Very Good**	**Good**	**Fair**	**Poor**
	◉	○	○	○	○

Research Grants Council 研究資助局

General Research Fund and Early Career Scheme

VIEW COMMENTS FROM EXTERNAL REVIEWER 3

Project Number : 17115422
Project Title : Combined Effects of Communicating Genetic Risk of Type 2 Diabetes and Wearable Technologies On Objectively Measured Behavioral Outcomes in Overweight or Obese East Asian Individuals.
PI Name : Dr Kim, Youngwon

Section A : Detailed Comments

1. Please comment on the objective(s) of the proposal, and whether the research agenda adequately addresses the objective(s)?

Excellent	Very Good	Good	Fair	Poor
○	●	○	○	○

Comments:

The proposal has very clear objectives and the research design could address these objectives

2. Please comment on the Research Design and Methodology.

Excellent	Very Good	Good	Fair	Poor
○	●	○	○	○

Comments:

1. The samples from e-mail or flyers. However, the neighborhood environmental characteristics of samples may influence an individual's PA and behaviors, such as proximity to parks or gyms. Should it be incorporated in the sampling design?
2. This proposal focuses on at-risk populations. However, according to the hypothesis of the proposal, combining wearable devices is assumed to promote an individual's PA. It may have similar effects on both at-risk and non-at-risk populations. Is it more critical to compare the difference of effects of wearable devices between at-risk and non-at-risk populations?

3. Please comment on the feasibility of the proposed research.

Excellent	Very Good	Good	Fair	Poor
○	●	○	○	○

Comments:

The research is feasible.

4. What do your consider to be the most original or innovative aspect of the proposed research? What advances would the research result bring about to the related field if the proposed research is successful?

Comments:

The proposal will clarify the effect of the integrated use of individuals' own genetic makeup and wearable devices have considerable potential to motivate overweight or obese East Asian individuals to initiate and sustain a more physically active lifestyle.

5. Please comment on the reasonableness of the proposed budget and manpower planning and project duration.

Comments:

The budget and manpower planning are reasonable.

6. Overall Comments

The proposal will clarify the effect of the integrated use of individuals' own genetic makeup and wearable devices have considerable potential to motivate overweight or obese East Asian individuals to initiate and sustain a more physically active lifestyle. However, there are two following points that may be considered further.

1. The samples will be from e-mail or flyers. However, the neighborhood environmental characteristics and social-economic status of samples may influence an individual's PA levels and behaviors, such as proximity to parks or frequency of going to gyms. Should these factors be incorporated in the sampling design and statistical models?
2. This proposal focuses on at-risk populations. However, according to the hypothesis of the proposal, combining wearable devices is assumed to promote an individual's PA. It may have similar effects on both at-risk and non-at-risk populations. From a health promotion perspective, is it more critical to compare the difference of effects of wearable devices between at-risk and non-at-risk populations?

Section B : Summary of Assessment

The project :

**Scientific/scholarly merit**	Excellent	Very Good	Good	Fair	Poor
	○	●	○	○	○
**Duration Proposed**	Too Long	Appropriate	Too Short		
	○	●	○		
**Impact of Research**	High	Moderate	Low	None	
	○	●	○	○	

The principal investigator :

**Ability to undertake the proposal**	**Excellent**	**Very Good**	**Good**	**Fair**	**Poor**
	○	◉	○	○	○
**Track record in field**	**Excellent**	**Very Good**	**Good**	**Fair**	**Poor**
	○	◉	○	○	○
